# Do Income, Race and Ethnicity, and Sprawl Influence the Greenspace-Human Health Link in City-Level Analyses? Findings from 496 Cities in the United States

**DOI:** 10.3390/ijerph15071541

**Published:** 2018-07-20

**Authors:** Matthew H. E. M. Browning, Alessandro Rigolon

**Affiliations:** Department of Recreation, Sport and Tourism, University of Illinois at Urbana-Champaign, Champaign, IL 61820, USA; rigolon@illinois.edu

**Keywords:** cities, greenspace, tree cover, obesity, mental health, income, race, ethnicity, sprawl, moderation, health disparities

## Abstract

Examination of the greenspace—human health relationship operates in at least four dimensions: what is considered greenspace? which moderators and mediators are included? what outcomes are measured? and which units of analysis (e.g., individuals, cities) are studied? We examined three of these four dimensions in a cross-sectional study of 496 of the 500 most populated US cities (total population size = 97,574,613, average population per city = 197,920). Spatial average models tested the effect of two greenspace measures (Normalized Difference Vegetation Index greenness and tree cover) on two outcomes (obesity and mental health), while adjusting for income, race and ethnicity, sprawl, age, sex, physical inactivity, median age of housing, and total population. We conducted analyses at the city scale, which is an understudied unit of analysis, and compared findings to individual- and neighborhood-level studies. In two of four models, greenspace was associated with better health. We found race and ethnicity moderated this relationship with varying results. In full sample analyses, cities with greater percentages of non-Hispanic Whites showed links between higher tree cover and lower obesity but marginal relationships between higher greenness and lower obesity. In subsample analyses with majority-non-Hispanic Black cities, higher tree cover was associated with lower obesity and better mental health. These findings advance previous research by showing that race and ethnicity moderate the greenspace—health link at the city level.

## 1. Introduction

Policymakers and communities around the world almost universally recognize the benefits of urban greenspace for human health, wellbeing, and ecosystem services. Urban greenspace, referring to urban vegetation, including parks, gardens, yards, urban forests and urban farms—or more broadly—a vegetated variant of open space [1], has received increased attention. Numerous public health, urban planning, and environmental organizations in the US and around the world have recommended including large amounts of greenspace in cities and neighborhoods to improve human health and environmental sustainability [2]. Academics have also discussed urban greenspace and other natural environments “as a panacea”, or as an infrastructure investment that can cure almost all ills of urban regions if properly maintained [3].

An overly simplistic portrayal of greenspace as a universal cure for all health problems is problematic, because there are still many unknown aspects about the link between greenspace and health. A recent workshop of leading international experts conceded, “While the existing evidence affirms the beneficial impacts of greenspace on health, much remains to be learned about the specific pathways and functional forms of such relationships, and how these may vary by context, population groups and health outcomes” [4] (p. 301). Indeed, our critical analysis of academic research suggests that the association between greenspace and health varies widely in at least four dimensions: (1) what is considered greenspace; (2) which mediator or moderator variables are included; (3) which health outcomes are measured; and (4) which units of analysis are studied. Importantly, mediators and moderators can be a way to incorporate cultural and contextual factors in the study of the greenspace-health link, including race and ethnicity and characteristics of the urban fabric such as population density [4]. Further, moderators in general population studies have not been shown to be significant in other studies, such as those covering largely disadvantaged neighborhoods in densely urban areas [5]. In addition, results vary based on interactions between these four dimensions. For example, a city-level study of the impact of trees on obesity may show significant effects, but a neighborhood-level study of the impact of parks on mental health may not.

In this paper, we present a study that sheds light on how the greenspace—health link varies based on three of these four dimensions—greenspace, moderators, and health outcomes—and their interactions. We analyze whether income, race and ethnicity, and sprawl moderate the greenspace-health link for a sample of 496 of the 500 most populated cities in the US. We do not directly examine the fourth dimension by comparing units of analysis. Rather, we present results for an understudied unit of analysis with direct relevance to greenspace policy and funding (i.e., the city level) and compare our results to past research on other units of analysis (e.g., the individual level).

Our approach is motivated by the need to address important literature gaps, recent changes in funding for greenspace and health promotion, and major health equity issues in the US. First, we recognize the necessity to disentangle the complex findings on the impact of greenspace on health. Second, very little research on the greenspace-health link has used cities as the unit of analysis, and findings of one study suggested urban sprawl might moderate the impact of greenspace on health [6]. Third, recent changes in funding mechanisms for greenspace and health promotion in the US have led cities to compete against each other to secure competitive grants from federal, state, and nonprofit agencies [7,8,9,10]. As competitive grant mechanisms tend to favor more affluent cities [7,8,9], findings about the greenspace-health link at the city level can raise awareness among grant-makers of how their work impacts health and equity. Fourth, the US is experiencing dramatic health inequities, as low-income and ethnic minority people have consistently poorer health outcomes than affluent White people [11]. Given such health disparities, we focused particularly on income and race and ethnicity as moderators of the greenspace-health links in order to understand which income and racial or ethnic groups particularly benefit from higher provisions of greenspace.

### 1.1. Literature Review: Why Does the Greenspace—Health Relationship Vary?

#### 1.1.1. Different Types of Indicators of Greenspace Exposure

Scholars have provided a variety of definitions of “greenspace” [1], and emerging research suggests some types of indicators of greenspace exposure exhibit more consistent relationships with health than others. Land-cover databases and Light Detection and Ranging (LiDAR) imagery allow researchers to compare, for instance, the differing effects of forests versus grasslands on health [4]. These comparisons have been conducted in only a handful of studies, but have largely shown that trees and shrubs are associated with better health outcomes than grass [12,13,14]. Yet, despite the emerging evidence that trees drive the greenspace-health connection, most studies continue to use generalized “greenness” measures [15]. Scholars are beginning to demonstrate how generalized measures underestimate both tree cover and small patches of greenspace [16,17].

#### 1.1.2. Different Factors Moderating the Greenspace—Health Link

Mediators and moderators can influence the strength and direction of the greenspace-health link [18]. Variables likely to affect the greenspace-health link in urban areas include contextual and cultural factors such as socioeconomic status (SES), race and ethnicity, and sprawl. Several studies show that the greenspace-health relationship is *only* present in lower-SES areas or is stronger in low-SES areas than high-SES areas [4]. Examining SES as a moderator is critically important to determine the direction of the greenspace-health relationship. Ongoing research by Kuo, Browning and colleagues suggests that the direction of greenspace impacting children’s cognitive performance reverses, from an inverse to a direct association (a change from negative to positive Beta coefficients), when SES is included as a moderator rather than only a confounder [19]. Many studies have used income and/or education to model SES moderation, but very few have examined race or ethnicity [20,21]. This is a particularly glaring omission because ethnic minority people in the United States have poorer health outcomes and healthcare access than the more affluent, White population [11].

Another hypothesized but understudied moderator in greenspace-health literature is urban sprawl. Sprawl, describing the extent to which urban areas have decentralized structures and low population densities [22,23], was implicated in at least one of the greenspace health studies with unexpected findings. Richardson and colleagues [6] suggested that greener cities have higher rates of all-cause mortality than less green cities because the former tend to be more spread out, which requires greater car use and leads to unhealthy lifestyles [24]. To our knowledge, no study has since tested whether sprawl moderates the greenspace-health relationship, but research has shown that sprawl, in general, is associated with poorer health outcomes [25,26] and more greenspace [6].

#### 1.1.3. Different Health Outcomes

The health and wellbeing outcomes selected for measurement also influence greenspace-health study findings. For example, dozens of studies found that greenspace is consistently associated with lower all-cause mortality [27], while others showed mixed results for asthma and allergies [28]. Myriad more outcomes (e.g., cardiovascular disease, cancer, and diabetes) lack a sufficient literature base to draw conclusions [15,16,29].

Obesity and mental health have been particularly well-studied but have yielded mixed results. Some reviews highlighted that greenspace is linked to reduced mental illness [29,30], whereas several other reviews concluded that greenspace has a limited impact on mental health, or that the findings are mixed [15,31,32]. Regarding obesity, authors reported a consistent link between greenspace and obesity in one review [33], mixed findings in three others [15,16,29], and no associations in a fifth [32].

#### 1.1.4. Different Units of Analysis

Lastly, greenspace-health findings might vary based on the unit of analysis, for example, individuals, neighborhoods, and cities. In particular, results are mixed when the unit of analysis covers a large geographic area, such as a city. Richardson and colleagues focused on 49 large US cities and found no relationship between urban greenspace and mortality related to heart disease or diabetes [6]. The authors found a positive relationship between green land cover and all-cause mortality, suggesting that greener cities have higher mortality rates. West and colleagues found that the density of parks (per 1000 residents) in the 85 largest US cities was correlated with higher levels of citywide physical activity and lower levels of citywide obesity [34]. A later study with 44 US cities highlighted that only park quantity (percentage of city area covered by public parks) correlated with citywide wellbeing levels while park quality (spending per capita on parks) and park access (percentage of people within 0.5 mile of parks) did not [35]. In summary, out of three articles that examined greenspace-health across cities, four analyses found that greenspace was not associated with better health, one found greenspace was associated with worse health, and only two associated greenspace with better health.

Analyzing the greenspace-health link at the city level is important due to recent changes in how greenspace and other health promotion initiatives are funded in the US. Over the last several decades, US cities have increasingly shifted their mechanisms for funding the construction and improvement of urban greenspaces, from tax-based city funds to competitive grants provided by state and federal agencies [7,8,9]. As many US cities have reduced or frozen their property taxes, which has led to fewer resources for urban greenspaces, nonprofits, state agencies, and the federal government have provided a variety of grants to fill these funding gaps [7,9]. Yet, competitive grants can aggravate existing inequities in greenspace provision [8,36,37]—wealthier cities are more likely than lower-income cities to have the skills and capacity to prepare competitive grant applications [7,8,9]. Furthermore, greenspace is increasingly framed as a health promotion tool [38], and the majority of funding to local public health departments (at a county level) leading health promotion initiatives flows from the federal government through competitive grants administered by agencies such as the US Department of Health and Human Services and Centers for Disease Control and Prevention [10]. Thus, funders of greenspace and health promotion initiatives would benefit from understanding cross-city differences in greenspace benefits.

### 1.2. The Current Study: Examining the Greenspace—Health Link Across Cities

In this study, we analyzed how types of indicators of greenspace exposure, moderators, and health outcome impact the greenspace-health link at the city level. We examined how two measures of greenspace–Normalized Difference Vegetation Index (NDVI) greenness and tree canopy cover—relate to two health outcomes—obesity and mental health. In an attempt to boost power and overcome limitations of past analyses at the city level, we used a larger sample than previous analyses (*n* = 496). We also examined the impact of likely moderating factors (income, race and ethnicity, and urban sprawl) on the greenspace-health relationship to understand how the benefits of greenspace vary by demographic and urban fabric characteristics.

We posed two overarching research questions: First, what is the relationship between citywide greenspace and obesity and mental health? Second, how do income, race and ethnicity, and urban sprawl moderate the impact of greenspace on health at the city level? Based on the literature reviewed above, we hypothesized that greener cities have lower rates of obesity and better mental health, and the beneficial impacts of greenspace are stronger in more disadvantaged cities (higher shares of racial-ethnic minorities or lower median household income) than in more affluent cities with a higher White population. We also hypothesized that greenspace is less beneficial for health in cities with higher levels of sprawl than in more compact cities.

## 2. Materials and Methods

### 2.1. Sample

**Figure A1:**
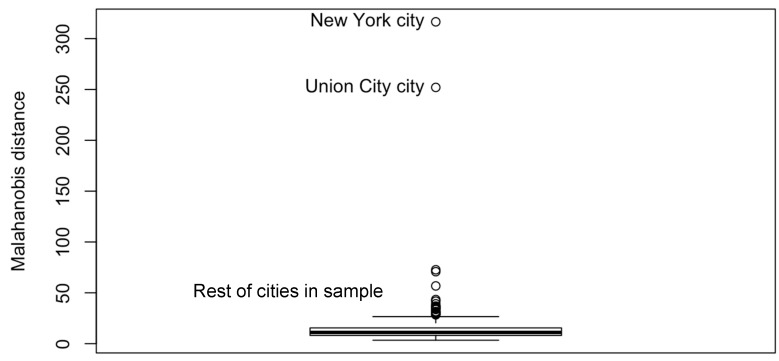
Visual inspection of the Mahalanobis Distance boxplot showed that New York, NY, USA, and Union City, NJ, USA, were hugely different than the other cities in our sample. Mahalanobis Distances were calculated with the following variables: *obesity, poor mental health, greenness, tree cover, median income, percent White, percent drivers, population density, median age, percent female, physically inactive, median age of housing, and population*.

We drew our sample from the 500 most populated cities in the United States. Two cities (Anchorage, AK, USA; Honolulu, HI, USA) did not have tree cover data as they were outside of the contiguous United States. After testing for multivariate normality using Mahalanobis Distances (MD), we visually identified New York, NY, USA, and Union City, NJ, USA, as outliers (See Appendix A, Figure A1). Without these outliers, our sample included 496 of the 500 most populated US cities. In total, these cities contained 97,574,613 residents in 2016. The average population per city was 197,920.

### 2.2. Sources of Data and Measures

We obtained health data from 2017 release of the Centers for Disease Control and Prevention (CDC) 500 Cities Project [39]. The CDC generated small area estimates (census tract and city-wide) of health by linking geocoded health surveys and high spatial resolution population demographic and socioeconomic data, while accounting for associations between individual health outcomes, individual characteristics, and spatial contexts [40,41]. The data used in these estimates is from the CDC’s Behavioral Risk Factor Surveillance System (BRFSS), which is based on phone interviews conducted with more than 400,000 adults each year in all 50 states [41]. The BRFSS recruitment methodology has been refined since this survey’s initial launch in 1984 and is now considered a gold standard in telephone-based health surveys [42]. As the median nationwide response rate is around 47%, the total number of respondents is close to 200,000 individuals [43]. A 2014 meta-analysis comparing nationwide BRFSS data to other health data confirmed that the BRFSS measures included in the current study were moderately to highly reliable and valid [44]. The 500 Cities Project data have also been used in at least four peer-reviewed articles, largely in medical journals, since their release approximately 1.5 years ago [45,46,47,48].

City-level estimates for health outcomes from the 500 Cities Project have very small margins of errors, while tract-level values have much larger errors due to the smaller sample size [39]. For example, for Birmingham, AL, USA, the margin of error at the city-level for percent poor mental health is ±0.25% (the estimate is 17%). For the same variable in Birmingham, the margin of error for census tracts is, on average, ±1.65% and as high as ±2.9%. Thus, for certain census tracts, estimates of percent poor mental health varied between 14.9% and 20.7%. Given these relatively large margins of errors for census tract estimates, and given the aforementioned changes in funding for greenspace and health promotion, we decided that a city-level study would be more rigorous and compelling.

We focused on two health measures for respondents aged 18 and over in each city: obesity and poor mental health. Obesity refers to the percentage of people who had a body mass index (BMI) larger than or equal to 30.0 kg/m^2^ as calculated from their self-reported weight and height. Pregnant women and respondents reporting extremely high or extremely low values for height and weight were excluded from this calculation. Poor mental health describes the percentage of people who reported that their mental health was not good in 14 or more of the past 30 days. Both obesity and mental health measures were, therefore, binary variables during data collection (obese-levels of BMI or not; good or not good mental health). When aggregated to the city-level, they became continuous variables (percentages of obese residents and percentage of residents with poor mental health). Among the various health measures reported in the 500 Cities Project dataset, we chose obesity and poor mental health because we were interested in studying health outcomes for which the greenspace-health link is well studied but with mixed findings (see Section 1.1.3). In particular, we aimed to ground our study in a substantial body of literature to uncover, for obesity and mental health, how the type of indicator of greenspace exposure, the unit of analysis, and moderators influenced the greenspace-health link.

Greenspace data were drawn from two sources to compare between overall “greenness” and tree cover. Greenness was obtained from 250 m NASA’s Moderate Resolution Imaging Spectroradiometer (MODIS) Vegetation Indices [49]. We calculated the Normalized Difference Vegetation Index (NDVI) from MODIS imagery in the summer months with the most leaf cover (June and July). NDVI shows the density of “greenness” and is calculated with the visible and near-infrared light reflected by vegetation. NDVI typically ranges from −1.0 to 1.0 where −1.0 represents complete cover by water, snow, ice, or rock and 1.0 represents complete cover by healthy green vegetation. We multiplied these values by 100 to create a range more similar to the scales of other variables. Tree cover was obtained from the most recently available nationwide dataset of tree canopy cover at moderately high resolution: the US Forest Service Percent Tree Canopy layer provided by the Multi-Resolution Land Characteristics Consortium in their 2011 National Land Cover Database. These 30 m resolution data used standardized preparation, classification, and quality control protocols to develop a percentage (from 0 to 100%) of tree cover in 65 distinct mapping zones for the continental US from Landsat-5 and Landsat-7 imagery. The resulting data have an average error ranging from 6% to 17% [50], although underestimates are more common in urban than rural areas [51]. Citywide greenspace values for both greenness and tree cover were the mean value of all overlapping pixels within municipal boundaries provided by the US Census Bureau’s Topologically Integrated Geographic Encoding and Referencing system [52]. These calculations were performed with the zonal statistics tool in ArcGIS 10.5.1 (ESRI, Redlands, CA, USA).

Covariate data, including those for possible moderators, were collected from the US Census Bureau’s American Community Survey (ACS) for 2012–2016 (5-year estimates) [53]. Each year, the US Census Bureau surveys approximately two million US residents to gauge the latest demographics, housing, and employment information. To minimize margins of errors, data for geographies with less than 65,000 people are aggregated for 5-year periods (e.g., 2011–2016). Although 5-year estimates for small geographies, such as block groups or census tracts, have shown very large margins of errors in some circumstances, estimates for larger geographies such as cities have acceptable margins of error [54]. We collected ACS data for a number of variables describing urban sprawl and socio-demographics factors. For sprawl, we considered *population density* (people per acre), *residential density* (housing units per acre), and *percent drivers* (percentage of people who drive to work alone). *Percent drivers* is associated with measures of sprawl, such as population density [22,23], as more decentralized and less densely populated cities make public transit less economically viable and likely require more residents to commute via car; therefore, *percent drivers* has been used as a proxy for sprawl [8,55]. We considered cities with either a high percentage of driving commuters or a higher density as being characterized by higher urban sprawl. We found that *population density* and *residential density* were highly correlated (*r* = 0.94) and, further, that including both measures in initial multivariate models resulted in multicollinearity—Variance Inflation Factor (VIF) values for both variables were over 14.0. Because *population density* was more strongly correlated with health outcome variables, we did not use *residential density* as a predictor in multivariate analyses but used it instead as one of the moderators (see Section 2.3). In contrast, *percent drivers* showed modest correlation with *population density* (*r* = −0.63) and including both in multivariate models did not cause multicollinearity. Therefore, both were used in the reported analyses below.

Key demographic variables describing income and race and ethnicity include *median income* and *percent White*. *Median income* describes the citywide median household income values expressed in US dollars, which is a key descriptor of a city’s socioeconomic status. *Percent White* describes the percentage of non-Hispanic White residents. In the US, non-Hispanic Whites are the largest racial-ethnic group, making up 61.3% of the total population in 2016 [53]. Throughout the paper, we use the phrase “race and ethnicity” rather than one of the two terms because *percent White* includes elements of both: White (characterized as race) and non-Hispanic (characterized as ethnicity). Over the course of US history, structural racism against racial and ethnic minority people (e.g., non-Hispanic Blacks and Hispanics/Latinos) has led to health inequities, with non-Hispanic Whites experiencing significantly better health outcomes [56]. Such health inequities, combined with uneven greenspace provisions that also put non-Hispanic Whites at an advantage [36], warrant the use of *median income* and *percent White* as moderators of the greenspace-health link.

Other covariates include *percent female* (percentage of female residents), *median age* (median age of city residents), *total population* (number of people permanently residing within city limits), *percent degree* (percentage of people aged 25 and older with a bachelor degree or higher), and *median age of housing*. We used *median age of housing* to model a city’s development timeline: Cities with older residential buildings were likely founded earlier than those with newer residential buildings. Different development timelines might impact the provision of greenspace, as older cities were planned with walkable pocket parks, while newer cities tend to contain larger but less diffuse greenspaces that are intended to be accessible via car [8]. Lastly, *percent inactive* was drawn from the CDC 500 Cities Project. This measure was the percentage of BRFSS respondents who answered “no” to the following question: “During the past month, other than your regular job, did you participate in any physical activities or exercises such as running, calisthenics, golf, gardening, or walking for exercise?” Table 1 summarizes the variables included in this study.

Datasets were obtained for the closest possible years of overlap. The 2012–2016 ACS data can be considered as an average of 2014 demographic data, which is the temporal midpoint between the two extreme years of data collection. The midpoint matches with the CDC data, which was collected in 2014 and 2015. MODIS data for NDVI-derived greenness were collected during the final year of ACS data, while NLCD tree cover data were collected for the most recent year available (2011). The moderate-resolution remote sensing measures of greenspace coverage do not seem to change much in a 5-year time period, even in cities with intensive urban greening efforts [57].

### 2.3. Analyses

We first performed bivariate analyses to test associations between health, greenspace, and covariates. We used Pearson product correlations at an alpha level of *p* < 0.05. Next, we created multivariate models with each health and each greenspace variable while adjusting for covariates and spatial patterns in the data. Each model included one dependent variable (obesity or poor mental health), one independent variable (greenness or tree cover), and all covariates. Initial analyses with ordinary least squares (OLS) regression showed high variance inflation factor (VIF) values for *percent degree* and *median income*. Because the latter was more highly correlated with health and greenspace, *percent degree* was removed from subsequent models. This solved multicollinearity concerns and all subsequent model VIF values were 3.5 or lower. OLS models also showed that spatial autocorrelation was present in model residuals (Moran’s I for *obesity* and *poor mental health* models were statistically significant, *p* < 0.001). In other words, cities that were closer together shared more similar demographic, health, and greenness characteristics than cities farther away, which, if not corrected for, introduced non-random bias in beta coefficients of regression models.

Given the potential for spatial autocorrelation issues, we used more advanced spatial models. We first ran the LaGrange Multiplier Test to determine which spatial regressions were most likely to resolve spatial autocorrelation concerns [58]. We then tested how the model fit, as measured by AIC (Akaike Information Criterion), and spatial autocorrelation, as measured by Moran’s I, varied between models. We found that spatial moving average (SMA) models showed moderately higher (poorer) model fit values than some other spatial regression techniques but also non-significant Moran’s I values in greenspace-health models (see Appendix A, Table A1), which indicated that spatial autocorrelation effects were resolved. SMA models integrate a kernel function that smooths out random noise across a geographic space while preserving the underlying covariance function [59]. We thus report our findings based on the SMA models. The comparative model fits, rather than effect sizes or variance, are explained in these models because SMA models use maximum likelihood estimates and introduce kernel smoothing. However, the coefficients for significant greenspace-health relationships are somewhat consistent between models, and OLS models suggested that each 10% increase in greenspace was associated with a 0.2% decrease in obesity, before adjusting for spatial effects.

We then performed moderation tests to examine how the greenspace-health relationship varied across levels of income, race and ethnicity, and sprawl. Flips in the direction of the association between two variables are possible from both moderation and mediation effects, and both effects likely play roles in the greenspace-health relationship. For many epidemiological studies, moderation is more suitable for statistical testing [4]. Mediation implies that there is a cause-and-effect relationship between the independent variable of interest (i.e., greenspace) and the mediator (i.e., income), whereas moderation does not require a causal relationship [60]. The greenspace-health literature is dominated by cross-sectional studies such as the current study. Claiming that one variable caused a change in another is therefore not possible [15,29]. For this reason, we ran moderation tests rather than mediation tests.

In this study, we tested the moderating effects of five possible variables (*median income*, *percent White*, *population density*, *residential density*, and *percent drivers*) in each of the four greenspace-health main effect models. We centered and standardized greenspace and the moderators, and we added interaction terms to each model. Statistically significant interaction terms indicated that moderation was present. We then plotted the greenspace-health relationships for the moderators that were significant using line graphs at three values of the moderator: the mean, −1 standard deviations from the mean, and +1 standard deviations from the mean. These plots allowed us to visually examine how moderators impacted the slope and direction (direct or inverse) of the relationship between greenspace and health. Although not central to the research questions of the current study, we also considered *physical inactivity* as a potential moderator because exercising in greenspace can improve health outcomes [4]. However, because we found physical inactivity had no moderation effects on greenspace and *obesity* or *poor mental health* (data not shown), we do not report any further description of its moderation impact.

Because greenspace provision differs for cities with different racial-ethnic composition [8], we examined how the greenspace-health relationship varies based on a city’s largest racial-ethnic group. First, as explained above, we tested the percentage of non-Hispanic Whites as a moderating variable in analyses with our full sample (*n* = 496). Second, we tested for significant relationships between greenspace and health outcomes in smaller numbers of cities based on racial and ethnic composition. We split our sample into cities with a non-Hispanic Black majority (*n* = 44), a Hispanic or Latino majority (*n* = 103), or a non-Hispanic White majority (*n* = 349). “Majority” was defined by which group had the largest percentage, not which group was 50% of the population or more. Thus, a city like Phoenix, AZ, which has 44% non-Hispanic White and 42% Hispanic, was classified as majority-non-Hispanic White. In summary, we conducted analyses of race-ethnicity with four samples of cities: (1) the entire set of 496 cities; (2) the 44 cities with a non-Hispanic Black population majority; (3) the 103 cities with a Hispanic or Latino population majority; and (4) the 349 cities with a non-Hispanic White population majority.

Analyses were performed using the R statistical software program Version 3.4.2 (R Foundation for Statistical Computing, Vienna, Austria) and RStudio Version 1.1.383 (RSTudio Team, Boston, MA, USA). Datasets were merged through a unique identifier provided for cities by the US Census Bureau. Supplementary material associated with this article includes a programming script for R Studio Markdown, which details how we merged data and ran analyses included in this paper.

## 3. Results

### 3.1. Descriptive Statistics

**Figure 1:**
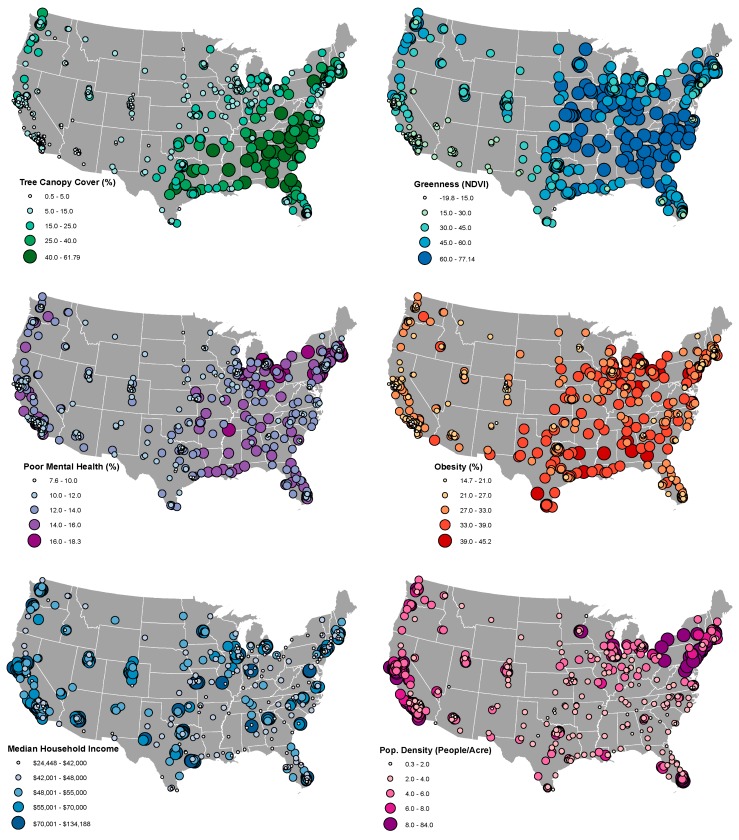
Variation in key health, greenspace, and moderating factor levels in cities across the United States.

Our sample of cities varied widely across all variables of interest (Table 2). *Obesity* ranged from less than 15% in four California cities (Fremont, Irvine, Milpitas, and San Ramon) to more than 45% in two midwestern cities (Gary, IN, USA and Detroit, MI, USA). *Poor mental health* ranged from less than 8% in Plymouth, MN, USA and Sugar Land, TX, USA to more than 18% in New Bedford and Fall River, MA, USA. *Greenness* levels were below zero in two cities with substantial amounts of surrounding water or rock cover (San Francisco and Alameda, CA, USA) and over 75 in cities with particularly high amounts of leafy green vegetation cover (e.g., Athens, GA, USA and Nashville, TN, USA). *Tree cover* was lowest in North Las Vegas, NV, USA (<1%) and highest in Sandy Springs, GA, USA, and Gainesville, FL, USA (>60%). The distribution of these variables clustered geographically across the US (Figure 1). *Tree cover* and *greenness* were generally higher in the East, *obesity* was higher in the East and the South, *poor mental health* was higher in the Northeast, and *median income* and *population density* were higher on the eastern and western coasts.

### 3.2. Bivariate Correlations

Bivariate correlations showed that greenspace measures and all covariates, with the exception of *population*, were significantly related to health (Table 3). Surprisingly, correlations highlighted that greener cities had *more* obesity and *worse* mental health on average before controlling for confounders. Cities with higher income, education, and proportion of White residents had less obesity and better mental health, which reflects well-known health disparities in the US [56]. Cities with younger residents, newer housing, more females, and more physically active residents also showed better health. Sprawl measures were related to *obesity* but not to *poor mental health*. Cities with more people who drove alone to work or had lower population densities had higher rates of obesity. Despite *population* not being correlated with either health outcome in bivariate correlations, we retained it in multivariate analyses because of the large range of population sizes in our sample.

### 3.3. Effects of Greenspace on Health in Multivariate Models

In contrast to bivariate correlations, multivariate models generally showed some evidence that greener cities had *better* health outcomes than less green cities after adjusting for spatial autocorrelation and confounders (Table 4). Two of four models (*poor mental health*~*greenness* and *obesity~tree cover*) showed statistically significant relationships between higher greenspace and better health (*p* < 0.01 and *p* < 0.05, respectively). No statistically significant relationships were found for the relationship between greenness and obesity or tree cover and mental health.

### 3.4. Moderation Effects in the Greenspace—Health Relationship

When interaction terms between possible moderators and greenspace were added to models, we found evidence that race and ethnicity influenced the greenspace-health relationship. *Percent white* moderated the *obesity~greenspace* relationship in models with either tree cover or greenness as the greenspace measure (*p* < 0.001). There was also marginal support for *percent white* moderating the *poor mental health~tree cover* relationship (*p* = 0.099). No significant interaction terms were found for sprawl measures (*percent drivers*, *population density*, and *residential density*). Finally, we found marginal support for *median income* moderating the relationship between *tree cover* and *obesity* (*p* = 0.080).

**Figure 2:**
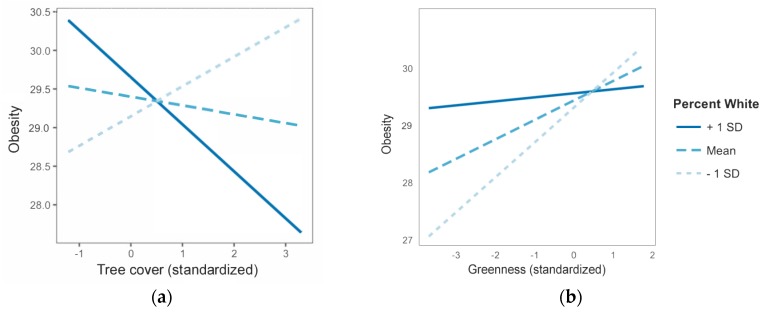
Interaction between percent non-Hispanic White residents in a city and (**a**) tree cover and (**b**) overall greenness on obesity rates. Findings are shown for our full sample of cities. *Solid blue lines* show the greenspace-health relationship for cities with one standard deviation *greater* than the mean regarding percentage of non-Hispanic Whites. *Light blue dotted lines* show the same relationship for cities with one standard deviation *less* than the mean. Importantly, subsample analyses suggest majority-non-Hispanic Black cities show the opposite effect as the full sample results shown in this figure; in majority non-Hispanic Black cities, there is a *positive* (not negative) association between health and greenspace (results not depicted in figures but reported in the text).

Moderator plots suggested that the direction of the greenspace-health relationship (negative versus positive correlation coefficients) can flip according to the racial-ethnic composition of a city (Figure 2). In our full sample, we found that in cities with greater percentages of non-Hispanic Whites, higher tree covers correlated with lower obesity rates. The opposite was true in cities with smaller percentages of non-Hispanic Whites: Higher tree cover correlated with *higher* obesity rates (Figure 2a). Overall greenness measures also showed a positive relationship between greenspace and obesity in cities with smaller percentages of non-Hispanic Whites. Cities with greater percentages of non-Hispanic Whites showed a shallow but positive relationship between *greenness* and *obesity* (Figure 2b).

Further investigation into race and ethnicity as moderators in subsamples of racial-ethnic majority cities showed different results than moderation tests with the full sample of 496 cities (Table 5). In majority-non-Hispanic Black cities (*n* = 44), more *tree cover* and *greenness* were significantly associated with lower obesity rates (*p* < 0.001 and *p* < 0.05, respectively). Models with majority-non-Hispanic White (*n* = 349) or majority-Hispanic (*n* = 103) cities were unreliable due to spatial correlations in residual terms (data not shown), so we could not make conclusions about the greenspace-health relationship in these types of cities.

A few exemplary cities provide a useful context for these findings. In support of full-sample analyses, Atlanta, GA, USA, has relatively high tree cover (52%) but low percentages of non-Hispanic Whites (37%) and higher than average obesity levels (32%). Cary, NC, USA, has nearly as much tree cover (48%) but a much higher percentage of non-Hispanic Whites (64%) and below average obesity levels (20%). Similar trends were seen in cities with low levels of tree cover. Both Chandler, AZ, USA, and San Bernardino, CA, USA had extremely low tree cover (2%). Chandler had a high percentage of non-Hispanic Whites (59%) and lower-than-average obesity rates (24%) while San Bernardino had a low percentage of non-Hispanic Whites (16%) and higher-than-average obesity rates (35%). These findings reflect the moderating effects of race and ethnicity on greenspace and health seen in Figure 2.

## 4. Discussion

In this study, we shed light on the complex associations between urban greenspace and health by analyzing how two measures of greenspace (NDVI-derived greenness and tree canopy cover) related to two health outcomes (obesity and mental health) at the city level, and how contextual and cultural factors such as income, race and ethnicity, and urban sprawl might moderate the greenspace-health link. We found some evidence for our hypothesis that greener cities would have less obesity and better mental health. In one model that controlled for spatial autocorrelation and adjusted for a range of confounding variables, higher levels of greenness were significantly related to better mental health. In another, higher tree cover was significantly associated to lower obesity rates (see Results Section 3.3).

Through moderation analyses, we found conflicting evidence about whether greenspace brings more health benefits to cities with large shares of racial-ethnic minority residents (see Results Section 3.4). Majority-non-Hispanic-Black cities showed beneficial relationships between greenspace and obesity, but across the entire sample, cities with a higher non-Hispanic White population showed more protective effects of greenspace on health than cities with fewer Whites.

These findings help unravel some of the mixed results that emerged in previous studies about the greenspace-health connection, since we conducted analyses with multiple measures of greenspace, three key types of moderators (income, race-ethnicity, and sprawl), two health outcomes, and an understudied unit of analysis. We describe the contributions to each of these dimensions below.

### 4.1. Understanding the Impact of Different Types of Indicators of Greenspace Exposure

In contrast to emerging literature that suggests trees and shrubs are more associated with better health than other greenspace measures, we did not find tree cover to be more strongly linked to positive health outcomes than greenness. However, NLCD-derived tree cover is known to underestimate coverage in urban areas [51]. It is possible that this measure also underestimates the effect size of tree cover on health, because it fails to adequately capture coverage in samples of cities. We also found evidence that the moderating effects of race and ethnicity impact the greenspace-obesity relationship more than the tree-cover—mental-health relationship. Examinations beyond greenspace type to greenspace quality, access, and funding may provide additional clarity on why we did not find trees to be more strongly linked with overall greenness in this study [35,36].

### 4.2. Understanding the Impact of Moderating Factors

Uncovering the impact of moderating variables can shed light on the importance of cultural and contextual factors on the greenspace-health link. Contrary to findings reported in a growing body of literature, we did not find that cities with lower median household incomes reap more health benefits from greater tree coverage than wealthier cities. Past research shows more deprived populations benefit more from greenspace in regards to obesity rates [61], mortality rates [61,62,63,64], birth outcomes [20,65,66], and insulin resilience levels [67]. The divergence of our results from these studies could be explained by differences in units of analyses, as most of such studies used individuals or neighborhoods as the unit of analysis [32], while we conducted a cross-city investigation. Importantly, lower SES residents are more dependent on greenspace in their neighborhood than more affluent people, as the former have less mobility outside of their neighborhoods than the latter [68,69]. As such, neighborhood-level analyses capture the actual quantity of greenspace that individual low-income residents might have access to on a daily basis, while city-level analyses do not. This could also help explain why our findings on SES as a moderator diverge from those of the previous literature.

The mixed findings of our study regarding the moderating effects of race and ethnicity confirm the importance of future research on this topic. Very few other studies have tested race or ethnicity as a moderator of the greenspace-health link, and those have shown mixed results [20,21]. In this study, we found that cities with fewer non-Hispanic Whites show *inverse* associations between greenspace and health (beta coefficients were negative). However, in a subsample of majority-non-Hispanic Black cities, we found associations between greenspace and health were in the opposite direction (beta coefficients were positive). Our full sample results may be explained by the fact that cities with higher shares of non-Hispanic Whites have more park coverage and parks with better programming than cities with more racial-ethnic minority people [8], which may lead to greater park “activation” by local residents and subsequent health benefits [70]. Our subsample analyses may be explained by regional differences in greenspace type and programming. The majority-non-Hispanic Black cities were concentrated in southeastern and eastern Atlantic states where greenspace provision is high, which constitutes a much narrower grouping of cities than in our full sample. Mixed results in our US sample may also be linked to interactions between concentrations of non-Hispanic Whites with other demographics. The percentages of non-Hispanic White and Hispanic residents were highly correlated (*p* = 0.70). Moderating effects may be driven by other unaccounted-for variables that correlate with non-Hispanic White percentages.

Regarding sprawl, our findings do not confirm the speculation made by Richardson and colleagues [6] that greener cities tend to have worse health because they are more sprawling. We found that sprawl does not have a significant moderating effect on the greenspace-health link, which suggests that tree cover is beneficial for obesity outcomes and overall greenness is beneficial for mental health outcomes regardless of a city’s population density. Richardson and colleagues made their speculation based on their findings showing associations between higher *mortality* rates and higher greenspace, and we examined *morbidities* related to mortality. Therefore, our study provides an initial test of the sprawl hypothesis and demonstrates the need for follow-up studies with the moderating effects of sprawl on *mortality*. If the sprawl hypothesis is indeed not supported, this finding would have strong implications for urban planners, who need to find strategies to implement greenspace infrastructure in both dense and sprawling cities.

### 4.3. Understanding the Impact of Health Outcomes

We build on a large body of literature suggesting that greenspace is linked to lower rates of obesity and better mental health. The greenspace-obesity link has been seen in both small-scale studies [71,72,73] and at least one larger-scale study [61]. This points to research showing that dense urban environments can promote physical activity [74]. We found that the impact of greenspace on obesity depends on the type of indicator of greenspace exposure used and the moderating effects of race and ethnicity. Our findings on greenspace and mental health were more robust. This is particularly encouraging, considering that cities can be stress-inducing places that adversely impact mental health [75]. A recent study with the same measure of poor mental health in metropolitan US counties found different qualities of greenspace distribution, for example, edges between forest and shrubland, predicted poor mental health differently [76].

### 4.4. Understanding the Impact of Units of Analysis

Our study suggests that greenspace can have a beneficial impact on mental health and obesity at the city scale. To date, nearly all greenspace-health studies in urban contexts have been at the individual or neighborhood level [32]. This study shows only null or beneficial associations between greenspace and health at the city scale. Mixed findings in past work [6,35] may be a symptom of inadequate control for sprawl or small samples sizes, two shortcomings that we directly addressed through the data selected and the research design used in this study.

### 4.5. Strengths and Limitations

Using cities as the unit of analysis is both a strength and a weakness of our study. As cities increasingly compete against each other for limited funding for greenspace and health promotion, grant-makers should understand the differentials in cities’ capacities to apply for grants [8]. This study is one of the few that shows how inequities vary across entire cities with regards to greenspace provision and health outcomes. Yet city-level measures do not reflect the greenspace available to residents on an everyday basis, particularly in larger cities. An additional strength is the relatively large sample of cities surveyed (*n* = 496). These cities covered broad geographical ranges and sociodemographic characteristics while adjusting for important confounders identified in past greenspace-health work and less-studied confounders related to building age, physical inactivity, and sprawl.

This study has limitations related to its cross-sectional ecological design and variable measures. Due to the cross-sectional design, causal inferences between greenspace and health cannot be made with confidence. We acknowledge that our findings may not translate to associations between individual city residents’ health and daily exposure to greenspace; concluding otherwise would be an ecological fallacy. Our intent was to test for observed associations at aggregated (population health) levels and provide hypotheses for testing in future investigations. Because of the importance of understanding city-scale associations between greenspace and health, the current study informs population-level understandings of greenspace and public health interventions using an appropriate ecological study design for this goal and provides hypotheses for further testing in future research. Another limitation is that health outcome measures were based on self-assessment only and did not include an objective health component. Also, the greenness measure (NDVI analyses of MODIS imagery) was at a coarse resolution (250 m), and pixels on the edges of cities capture ‘greenness’ measures in adjacent areas. In some cases, such as two California cities, this resulted in greenness values being negative because city limits abutted water features and seasonal wetlands. We did not examine climatic or vegetative differences across cities. Some cities cover large geographic areas (i.e., Jacksonville, FL, is over 875 square miles) and, especially in such cities, the greenspace-health relationships might vary by neighborhood. A final limitation regards residual confounding distortions, which are always a concern in epidemiological studies because they lead to biased findings and inflated or deflated effect sizes [4]. Fortunately, the impact of unmeasured confounders is often small and would only substantially bias the results if they were not correlated with other measured confounders [77].

### 4.6. Future Research

Replications of this study in cities in different countries—particularly those in the Global South, where relatively little research on this topic has been conducted [32]—would enhance our knowledge of the moderating effects of income, race and ethnicity, and sprawl. In particular, due to the positive impact of mixed land uses (i.e., housing near shops and services) on health [78], future research could analyze whether such variables can mediate the greenspace-health relationship at the city level. However, this would require the development of national datasets describing city-level land use mix. Also, future work could use higher resolution datasets, such as the University of Vermont urban Tree Canopy Assessments, which provide evaluations of where trees, shrubs, and grasses exist in select eastern and midwestern U.S. cities at a very fine scale (less than 1 m) [79]. Alternatively, future work could use emerging measures of greenspace access rather than simply quantity. For instance, the Trust for Public Land has developed the ParkServe dataset, which provides data on the percentage of people with walking access to parks [80]. Future investigations along these lines could focus on other health outcomes included in the CDC’s 500 Cities Project dataset, such the prevalence of cardiovascular diseases. Finally, studies about the greenspace-health link at the census tract level could complement the current investigation and determine to what extent our findings for cities scale down to neighborhoods. However, tract-level studies using CDC data to measure the greenspace-health relationships should include robust analyses of data uncertainty [81,82], because, as we noted, CDC data for this unit of analysis have relatively large margins of error.

## 5. Conclusions

In this study, we found some evidence that greenspace is positively associated with better health outcomes—specifically obesity and mental health—at the city level. Most importantly, our study advances previous research by showing that race and ethnicity moderate the greenspace-health link at the city level. In cities with larger shares of non-Hispanic White residents, greater tree cover was associated with lower obesity rates, while we found opposite results for cities with higher percentages of racial and ethnic minority people. However, in a subsample of majority-non-Hispanic-Black cities, we found positive associations between greenspace and health outcomes. By uncovering these complex findings, we contribute to the unraveling of how different types of indicators of greenspace exposure, moderators, health outcomes, and units of analysis impact the greenspace-health link.

The results of this study can inform policymakers, city planners, foresters, public health officials, and greenspace funding agencies about how the greenspace-health relationship varies by demographics and urban fabric. Citywide measures of tree cover may be associated with obesity prevention in cities with a higher non-Hispanic White population. If this association holds in future work, these findings pose challenges for the use of greenspace to address health inequities that negatively impact Hispanic and non-Hispanic Black residents in the US [56]. Indeed, if tree cover and greenness particularly matter for Whiter cities (with the exception of findings for majority-non-Hispanic-Black cities), planners, policy-makers, and public health officials seeking to address health inequities also need to implement other interventions, such as the provision of parks, recreation programs, and trails for active transportation. In addition, these interventions could target greenspace-deprived neighborhoods that also experience poor health outcomes. Thus, urban greening at the city level should also address those issues at the neighborhood level. Finally, because efforts to improve the provision of urban greenspace in low-income communities could result in green or environmental gentrification—which is the increase in rents and property values due to new green amenities—these efforts should use holistic approaches that, besides park creation and tree planting, also include the establishment and preservation of affordable housing [83,84].

## Figures and Tables

**Table 1:** Descriptive of variables and data sources.

Variable	Description	Source ^1^
Percent poor mental health (“poor mental health”)	Percentage of residents who reported poor mental health 14 or more out of the last 30 days	CDC
Percent obese (“obesity”)	Percentage of residents with Body Mass Index (BMI) ≥30.0 kg/m^2^	CDC
NDVI (“greenness”)	Mean of 250 m NDVI pixel values within city limit	MODIS
Percent tree cover (“tree cover”)	Mean of 30 m percent tree canopy cover pixel values within city limit	MLRC
Median income	Median household income in dollars	ACS
Percent degree	Percentage of residents aged 25 and older with bachelor, associates, professional, or doctoral degree	ACS
Percent White	Percentage of non-Hispanic White residents	ACS
Percent drivers	Percentage of people aged 16 or above commuting to work via automobile (alone) and a measure for urban sprawl	ACS
Population density	Number of residents per acre and a measure for urban sprawl	ACS
Median age	Median age of residents	ACS
Percent female	Percentage of female residents	ACS
Physically inactive	Percentage of people who did not participate in physical activities or exercise in the past month	CDC
Median age of housing	Median age of housing structure in years (2018 minus the median year when housing buildings were built)	ACS
Population	Total population of city	ACS

^1^ ACS is the American Community Survey run by the US Census Bureau. MODIS is Moderate Resolution Imaging Spectroradiometer. MLRC is Multi-Resolution Land Characteristics Consortium. CDC is Center for Disease Control and Prevention.

**Table 2:** Descriptive statistics.

Variable	Mean	Sd	Median	Min	Max
Poor mental health	12.4	2.1	12.4	7.6	18.3
Obesity	29.3	5.9	29.5	14.7	45.2
Greenness	45.5	17.7	47.9	−19.8	77.1
Tree cover	17.0	13.5	13.6	0.5	61.8
Median income	$56,515	$19,115	$50,956	$24,448	$134,188
Percent degree	31.9	13.9	29.3	6.0	76.7
Percent White	50.0	21.6	51.1	1.1	89.2
Percent drivers	76.1	9.1	78.5	28.2	89.9
Population density	6.4	4.9	4.9	0.3	34.0
Median age	35.0	4.1	34.8	22.6	47.6
Percent female	51.0	1.3	51.0	41.7	55.0
Physically inactive	25.8	6.2	26.0	13.0	44.8
Median age of housing	42.5	15.5	40.0	12.0	77.0
Population	197,340	297,127	110,983	42,556	3,918,872

**Table 3:** Bivariate correlations.

	(1)	(2)	(3)	(4)	(5)	(6)	(7)	(8)	(9)	(10)	(11)	(12)	(13)	(14)
Poor mental health (1)														
Obesity (2)	0.70 ***													
Greenness (3)	0.13 **	0.40 ***												
Tree cover (4)	0.18 ***	0.30 ***	0.72 ***											
Median income (5)	−0.80 ***	−0.76 ***	−0.24 ***	−0.20 ***										
Percent degree (6)	−0.76 ***	−0.60 ***	0.11 *	0.15 **	0.65 ***									
Percent White (7)	−0.30 ***	−0.19 ***	0.39 ***	0.16 ***	0.15 ***	0.43 ***								
Percent drivers (8)	−0.023	0.140 **	0.19 ***	0.07	0.023	−0.24 ***	0.17 ***							
Population density (9)	0.054	−0.21 ***	−0.37 ***	−0.31 ***	0.07	−0.019	−0.43 ***	−0.63 ***						
Median age (10)	−0.25 ***	−0.40 ***	−0.084	−0.013	0.41 ***	0.19 ***	0.24 ***	0.22 ***	−0.011					
Percent female (11)	0.26 ***	0.25 ***	0.25 ***	0.32 ***	−0.22 ***	−0.058	−0.045	0.12 *	−0.069	0.19 ***				
Physically inactive (12)	0.75 ***	0.86 ***	0.29 ***	0.28 ***	−0.72 ***	−0.64 ***	−0.39 ***	0.015	0.015	−0.33 ***	0.24 ***			
Median age of housing (13)	0.38 ***	0.23 ***	0.031	0.035	−0.32 ***	−0.16 ***	−0.15 ***	−0.51 ***	0.51 ***	−0.016	0.11 *	0.34 ***		
Population (14)	0.017	0.046	−0.085	−0.018	−0.077	0.026	−0.15 ***	−0.17 ***	0.12 **	−0.072	−0.018	0.056	0.083	

Text in gray indicates non-significant correlations, * *p* < 0.05, ** *p* < 0.01, *** *p* < 0.001.

**Table 4:** Four models testing the greenspace-health relationship at a city scale while adjusting for spatial and confounding effects.

Model Variables and Fit Statistics	Model 1	Model 2	Model 3	Model 4
	Obesity ^1^		Poor Mental Health	
	Greenness	Tree Cover	Greenness	Tree Cover
Greenspace variable	0.00037 (0.0090)	**−0.026** (0.11) *	**−0.0099** (0.0037) **	−0.0057 (0.0044)
Intercept	**7.3** (3.8) **^+^**	**6.6** (3.7) **^+^**	**3.6** (1.4) *	**3.6** (1.4) *
Median income ^2^	**−0.98** (0.18) ***	**−0.95** (0.18) ***	**−0.85** (0.071) ***	**−0.86** (0.072) ***
Percent White	0.0082 (0.0075)	0.011 (0.0071)	0.0014 (0.0030)	−0.00083 (0.0028)
Percent drivers	**0.033** (0.018) **^+^**	0.027 (0.018)	**0.013** (0.0068) **^+^**	**0.012** (0.0069) **^+^**
Population density	**−0.15** (0.033) ***	**−0.17** (0.034) ***	0.0085 (0.013)	0.010 (0.013)
Median age	**−0.11** (0.033) **	**−0.11** (0.032) ***	0.0025 (0.013)	0.0064 (0.013)
Percent female	**0.12** (0.072) **^+^**	**0.15** (0.071) *	**0.060** (0.027) **^+^**	0.043 (0.027)
Physically inactive	**0.65** (0.035) ***	**0.67** (0.034) ***	**0.21** (0.014) ***	**0.20** (0.014) ***
Median age of housing	**0.020** (0.011) **^+^**	**0.021** (0.011) **^+^**	0.0025 (0.0043)	**0.0064** (0.013) *
Population ^2^	−0.0042 (0.082)	0.00089 (0.081)	0.0022 (0.030)	0.0067 (0.031)
AIC	2155	2149	1242	1247
Lambda	0.77	0.78	0.96	0.95
Likelihood ratio test value	159 ***	168 ***	270 ***	271 ***
−2 log likelihood	2028	2124	1216	1212
Maximum likelihood residual variance	4.8	4.8	0.82	0.83
Moran’s I (observed)	−0.0045	−0.0081	−0.020	−0.019
Moran’s I (expected)	−0.0020	−0.0020	−0.0020	−0.0020

^1^ Unstandardized beta coefficients and standard errors shown for spatial moving average models, ^2^ standardized and centered so variables in the model were in a more consistent range, ^+^ *p* < 0.10, * *p* < 0.05, ** *p* < 0.01, *** *p* < 0.001.

**Table 5:** Findings from subsamples of cities with majorities of one race/ethnicity.

Subsample Characteristics		Green Space-Health Relationships Tested			
Majority Group	Number of Cities	Obesity-Greenness	Obesity-Tree Cover	Mental Health-Greenness	Mental Health-Tree Cover
Non-Hispanic Black majority	44	−0.054 *	−0.10 ***	n.s.	n.s.
Hispanic or Latino majority	103	n.s.	n.s.	n.s.	n.s.
Non-Hispanic White majority	349	n.s.	n.s.	n.s.	n.s.

* *p* < 0.05, *** *p* < 0.001.

## Supplementary Materials

The following are available online at http://www.mdpi.com/1660-4601/15/7/1541/s1, R Markdown code for running analyses and datasets.

## Appendix A

**Table A1:** Model fits and greenspace—health relationships across regression models.

Effects of Greenspaces on Obesity			
Model	Predictor Coefficient	Model Fit ^1^	
	*Greenness*	*AIC*	*Moran’s I*
Ordinary least squares (OLS)	**0.019 ***	2312	**0.17 *****
Spatial lag (SpL)	0.011	2240	**0.12 *****
Spatial moving average (SMA)	0.00037	2155	−0.0045
Spatial autoregressive (SAC)	−0.0060	2140	**0.044 *****
Spatial error (SpE)	−0.0060	2138	**0.044 *****
Spatial durbin error (SDE)	**−0.017 ^+^**	2110	**0.028 *****
Spatial durbin (SD)	**−0.021 ***	2107	**0.026 ****
	*Tree cover*	*AIC*	*Moran’s I*
OLS	−00.013	2315	**0.18 *****
SpL	**−0.020 ***	2237	**0.12 *****
SMA	**−0.026 ***	2149	0.50
SAC	**−0.032 ****	2132	**0.043 *****
SpE	**−0.031 ****	2131	**0.043 *****
SD	**−0.043 *****	2013	**0.028 *****
SDE	**−0.039 *****	2108	**0.032 *****
**Effects of Greenspaces on Poor Mental Health**			
**Model**	**Predictor Coefficient**	**Model Fit ^1^**	
	*Greenness*	*AIC*	*Moran’s I*
OLS	**−0.0097 ****	1510	**0.21 *****
SpL	**−0.0085 ***	1455	**0.16 *****
SMA	**−0.0098 ****	1242	**−0.020 *^2^**
SpE	−0.0022	1143	**0.051 *****
SAC	−0.0018	1140	**0.048 *****
SDE	−0.0010	1123	**0.032 *****
SD	−0.00012	1116	**0.021 ****
	*Tree cover*	*AIC*	*Moran’s I*
OLS	−00.00017	1517	**0.22 *****
SpL	−0.0026	1460	**0.17 *****
SMA	−0.0057	1247	−0.019
SpE	−0.0042	1142	**0.050 *****
SAC	−0.0035	1132	**0.043 *****
SDE	−0.00048	1120	**0.031 *****
SD	−0.0026	1112	**0.021 ***

^1^ Models are controlling for median household income, percent non-Hispanic White, median age for residents aged 25 and older with a bachelor degree or higher, percent female, total population, population density, housing age, percent physically inactive, and percent residents who drive alone to work; data are sorted by AIC value from largest to smallest; ^2^ *p* = 0.04, ^+^ *p* < 0.10, * *p* < 0.05, ** *p* < 0.01, *** *p* < 0.001.
